# Attitudes of health service providers: The perspective of people with disabilities in the Kumasi Metropolis of Ghana

**DOI:** 10.4102/ajod.v5i1.181

**Published:** 2016-08-16

**Authors:** Eric Badu, Maxwell P. Opoku, Seth C.Y. Appiah

**Affiliations:** 1 Department of Community Health, Center for Disability and Rehabilitation Studies, Kwame Nkrumah University of Science and Technology (KNUST), Ghana; 2 Department of Sociology and Social Work, Kwame Nkrumah University of Science and Technology (KNUST), Ghana

## Abstract

**Introduction:**

Awareness of disability issues has gained considerable interest by advocacy groups in recent years. However, it is uncertain whether attitudes and perceptions of all service providers and society have adjusted accordingly towards the health care of people with disabilities. This study sought to examine the attitudes of health providers from the perspective of people with disabilities in the Kumasi Metropolis.

**Methods:**

A cross-sectional study using semi-structured questionnaires was conducted with people with disabilities (with physical, hearing and visual impairments,) in the Kumasi Metropolis. The study used a multi-stage sampling involving cluster and simple random sampling to select 255 respondents split amongst the following five clusters of communities; Oforikrom, Subin, Asewase, Tafo and Asokwa. Data were analysed using STATA 14 and presented in descriptive and inferential statistics.

**Results:**

The study found that 71% of the respondents faced some form of discrimination including the use of derogatory remarks, frustration and unavailable required services on the basis of their disability, the type of services they need and the location. Women were 3.89 times more likely to face discrimination; Adjusted odds ratio (AOR) = 3.89 (95% confidence interval [CI]; 1.41, 10.76), and visually impaired was more likely to be discriminated at the facility compared with physical disability; AOR = 5.05 (95% CI; 1.44, 17.65). However, respondents with some educational qualification and those who stayed with their family members were less likely to face discrimination; AOR = 0.08 (95% CI; 0.01, 0.39).

**Conclusion:**

The study recommends the provision of in-service training for service providers to update their knowledge on disability issues and improve access to services for people with disabilities.

## Introduction

In most developing countries around the world, people with disabilities people with disabilities may feel reluctant to access health services although they may have serious health problems that require health service intervention (Shaikh & Hatcher 2005:51). This is because of their experiences from previous attempts to access health services, particularly the attitudes and perceptions of health care providers. Therefore, patients’ ability to accept and utilise services has a relationship with service providers’ attitudes (d’Ambruoso, Abbey & Hussein 2005; Jones *et al*. 2008; Shaikh & Hatcher 2005, 2007). The attitudes towards people with disabilities have been identified as stigma, for example, stereotypes, wrong perceptions and the use of abusive and discriminatory words (Iezzoni 2011). This may limit them from accessing health care compared with the non-disabled population. The stigma and use of abusive words is mostly experienced amongst women with disabilities and make them more vulnerable than their male counterparts (Aunos & Feldman 2002). Women with disabilities are most likely to experience stigma relating to marital and reproductive life. For instance, as a result of parenting difficulties, most service workers and families of people with disabilities, particularly with intellectual disabilities, strongly support sterilisation of females with disabilities despite the concerns over the years to ban involuntary sterilisation (Aunos & Feldman 2002).

Most health professionals may seem not to bother about people with disabilities at the health care setting and do not see the need to give them special attention. For instance, doctors in particular seem to have limited time to attend to patients and may not have special time for people with disabilities (Jones *et al*. 2008). This is most likely to happen in developing countries where there seems to be limited doctors attending to large number of patients. Most often than not, the genesis of the attitudes and perceptions towards clients with disabilities originates from the training institutions to the field of practice (Jones *et al*. 2008). Research has found that attitudes of health professionals are affected by low motivation, inadequate service providers and limited education on disability issues (Witter, Kusi & Aikins 2007). A study on the views of undergraduate physiotherapy students in Malawi found that whilst some students ‘empathised’ with people with disabilities, others revealed they felt uncomfortable when dealing with them (Amosun *et al*. 2013). The study therefore concluded that these negative attitudes and perception may have an effect on rehabilitation services that would be offered to people with disabilities when such medical students are attending them (Amosun *et al*. 2013). Another study on community-based rehabilitation (CBR) programme of the University of Philippines, Manila, found that the programme has improved the skills of disability-related issues, values and attitudes of the students. This improvement is, therefore, believed to reflect on their services as field practitioners (Magallona & Datangel 2012). Education of students in various institutions will improve their knowledge of stigma attached to issues of disability.

The studies by Amosun *et al*. (2013) together with that of Magallona and Datangel (2012) posit a strong case on the positive attitudes of health workers towards people with disabilities; yet they only focus on students. Singer (2012), however, found that school nurses face challenges when working with children with disabilities. Some of these challenges range from difficulty in communicating with the children, conducting health assessments and screening students. These challenges make nurses feel less comfortable and have negative attitudes when working with children with disabilities. The perception usually emerges because of a lack of experiences and limited knowledge on disability-related issues. White and Olson (1998:128) found that in an effort to ensure that nurses show positive attitudes towards people with disabilities, age, area of practice and length of practice are not important factors. However, evidence (Kroll *et al*. 2006) shows that educational level plays a major role to promote positive attitudes. In order to encourage positive attitudes towards people with disabilities, disability-related courses should be incorporated into the training curricula (Kroll *et al*. 2006). Field practitioners should also be equipped with disability knowledge and skills through in-service training. Findings by Flatt-Fultz and Phillips (2012) suggested that providing training for health service providers through videos may improve their knowledge on disability issues and change their attitudes.

Attitudes and behaviour of primary health care providers have been identified as a barrier for people with disabilities as they seek health care (Jones *et al*. 2008) and this has become problematic in developing countries including Ghana (Mensah *et al*. 2008). However, not much is known within the Ghanaian setting about the attitudes of health care workers towards people with disabilities. The results of such a study can be used to inform policy makers and other stakeholders. This article seeks to examine the attitudes of health care providers from the perspectives of people with disabilities in the Kumasi Metropolis in Ghana.

### Brief history of disability in Ghana

In Ghana, the recognition of people with disabilities started after the establishment of a rehabilitation unit at the 37th General Military Hospital in Accra between 1943 and 1947. The rationale of the programme was to reintegrate African soldiers with disabilities after the Second World War into the workforce. This programme was later handed over to voluntary service organisations in 1947. The government in 1950 absorbed the voluntary sector and took over the practical aspect of the work, leaving voluntary sectors to advocacy (Grischow 2011).

In 1960 a massive registration programme was, however, launched in the country after ‘John Wilson’ (Grischow 2011) estimated that 100 000 Ghanaians lived with some form of disability. This informed the government and led to the establishment of rehabilitation units and special education programmes to provide educational needs for people with disabilities all over the country. The rationale for this programme under first President Kwame Nkrumah’s administration was to integrate people with disabilities into the Ghanaian workforce. The work of organisations dealing with disability established in the late 1960s contributed greatly to the formation of many disability movements later and to the present day (Grischow 2011). Again, in an attempt to fight discrimination against people with disabilities as they seek employment, Legislative Instrument (632) Labour regulation was passed in 1969. The legislative instrument gave 0.5% quota to people with disabilities in all establishments. As part of this effort, offices were created in regions and districts to register and offer jobs to people with disabilities (The Danish Council of Organisations of Disabled People 2006).

### Prevalence of disability in Ghana

Worldwide, 15% of the population is estimated to live with some form of disability. This represents more than one billion people. The prevalence is higher in low income countries compared with developed countries (World Health Organisation [WHO] 2011). In every developing country, the prevalence is estimated at 10% to 15% (WHO 2013). This translates to about 2.4 to 3.6 million Ghanaians living with disability. However, the 2010 population census found that the prevalence of disability in Ghana is 3% which represents 737 743 people. The number of women with disabilities was 387 647 and that of males 350 096. The prevalence presented regional disparities where the Ashanti region had the highest rate of disability whilst Upper West had the lowest (Ghana Statistical Services [GSS] 2012). The rate of disability given by GSS both at the national and regional levels falls below the WHO estimate of 10% to 15% in developing countries. However, the high population of people with disabilities in the Ashanti region calls for more attention in research and policy circles.

## Study design and methods

The study used a cross-sectional design with quantitative method to examine the perspective of people with disabilities on health professionals’ attitudes towards their health care in the Kumasi Metropolis in Ghana.

### Study area

The setting of this study was the Kumasi Metropolis of Ghana, located in the forest zone covering a total land area of 254 square kilometres. The 2010 population and housing census found that the metropolis accommodates a population of 2 million people with an inter-censual growth rate of 5.4%. The metropolis has a total of 189 health facilities ranging from clinics to a teaching hospital. About 91% of the facilities in the Kumasi Metropolis are managed by private individuals. The metropolis has a doctor–patient and nurse–patient ratio of 1:41.606 and 1:7.866 respectively (GSS 2012). The rate of National Health Insurance Scheme (NHIS) registration in the metropolis is around 81% (GSS 2012; Kumasi Metropolitan Assembly [KMA] 2010) and makes health care arguably affordable in the metropolis. Over 60% of outpatient department attendance (OPD) are malaria cases making it a public health problem in the metropolis. It is, however, surprising that there is no available data on the number of people with disabilities who are enrolled into the NHIS each year (GSS 2012; Kumasi Metropolitan Assembly 2010; Mensah *et al*. 2008). The 2010 population census showed that the metropolis is divided into 10 sub-metros namely Asokwa, Asewase, Bantama, Suame, Manhyia, Oforikrom, Tafo, Nhyiaeso, Subin and Kwadaso (GSS 2012).

### Sample size and sampling

The sample size was calculated by considering the proportion of the population in the Kumasi Metropolis who have some form of disabilities. The proportion of people with disabilities in the study area is estimated at 2.6% based on the GSS 2010 census. The sample size was then calculated using this proportion of the population with significance level of 5%, allowing 0.03 degree of freedom, 10% non-response rate and design effect of 2. The total sample size was then estimated at 255 people with disabilities. The sample size was estimated using Cochran’s sample size formula:

$n_{0}=\frac{Z^{2}*(p)(q)}{d^{2}}$ where *z* = value (*z*): = 1.96 or 95% confidence level, *p* = prevalence of population, *d* = degree of freedom. (Cochran 2007; Naing, Winn & Rusli 2006)

The study used a multi-stage process involving cluster and simple random sampling to randomly selected communities in the Kumasi Metropolis. This method was applied because the study area had many clusters of communities. Firstly, the study randomly selected 5 clusters of communities (Oforikrom, Subin, Asawase, Tafo and Asokwa) out of 10 clusters based on definition of sub-metro in the metropolis. The second stage sampling involved selecting people with disabilities (with physical, hearing and visual impairments) using simple random sampling. A total of 255 people with disabilities were selected which was split equally amongst the five clusters of communities, that is, 51 people with disabilities were selected from each cluster.

The investigator and the research team used simple random sampling to select respondents. The research team zoned households and streets in the selected communities to identify prospective respondents. All people with disabilities were approached and the intent and procedures of the study were explained to them to enable them to freely decide whether or not to participate. People with disabilities were asked to pick from a box with ‘Yes’ and ‘No’ papers. All people with disabilities who picked ‘Yes’ in all the clusters, and consented, were enrolled. This was repeated to obtain the required sample size. The inclusion criteria was based on people with disabilities of 16 years and older, who stayed in the study area and accessed health care in the last 12 months within the metropolis.

### Data collection and analysis

The study administered a structured questionnaire to collect information from respondents. The questionnaire was developed in English but the interview was conducted in the respondents’ preferred dialect; English, sign language or Asante Twi. A professional interpreter volunteered to assist in the study. The data were collected over a period of two months (February to March 2014) to allow time to reach all participants. Each participant spent approximately 40 minutes to answer the questions. The main dependant variable was perceived attitudes of health service providers whilst the independent variables were issues related to attitudes, including discrimination and knowledge on disability issues. Result of the analysis was generated using descriptive and inferential statistics. Data were summarized into frequency and percentage. Tables and graphs were used to present the results. The data analysis involved the estimation of frequencies and percentages of background characteristics of respondents. The responses on the perceived attitudes of health professionals including discrimination, allowing time to ask questions and the rating of service providers’ knowledge on disability issues, were also presented in frequencies and percentages. Crude odds ratios and a 95% CI were calculated and followed by adjusted odds ratio. Respondents who did not experience discrimination when attempting to access health care were used as reference point for making comparison discrimination that people with disabilities received at health facility. Significance was set at p-value of less than 0.01 and 0.05. The data obtained from the field were first entered into Statistical Package for Social Sciences version 20 and transported to STATA version 14 for analysis.

## Ethical consideration

The study was conducted according to laws and regulations of the KNUST Committee for Human Research Publication and Ethics. The committee reviewed and approved the study protocols prior to the implementation of the study. A written informed consent was translated and explained to potential study respondents in a language well understood by them prior to their enrolment in the study.

## Results

### Background information

Table 1 presents the background characteristics of 255 people with disabilities who participated in the study. The study was conducted amongst three different disability groups namely physically-, hearing- and visually impaired persons in the Kumasi Metropolis. Out of 255 people with disabilities, 50.6% were men. The mean age of the respondents was 38 years. In terms of education, a little above one-third of the respondents (34.5%) reported they had no formal education, 16.9% completed tertiary education, 15.3% had senior high school education, 16.9% junior high school, 16.1% primary level and 0.4% had post-secondary education. The study further found that the majority of the respondents (28.6%) were unemployed whereas the remainder were split amongst government/civil servants (11.0%), trading (15.7%), farming (11.0%), apprenticeship (21.6%) and (12%) otherwise employed, including private business owners. The majority (81.5%) of the respondents were Christians. On the family status of the respondents, most respondents (85.1%) were staying with their family.

**TABLE 1:** Background characteristics of respondents.

Variable	Frequency	Percentage (%)
**Community of resident**		
Oforikrom	49	19.2
Subin	50	19.6
Asawase	51	20.0
Tafo	55	21.6
Asokwa	50	19.6
**Disability type**		
Physical disability	85	33.3
Visual impairment	85	33.3
Hearing impairment	85	33.3
Age*		
≤20	10	3.9
21–30	46	18.0
31–40	107	42.0
41–50	41	16.1
>50	51	20.0
**Gender**		
Male	129	50.6
Female	126	49.4
**Employment**		
Government/Civil servant	28	11.0
Trading	40	15.7
Farming	28	11.0
Apprenticeship/Craft	55	21.6
None	73	28.6
Other	31	12.2
**Educational level**		
No formal education	88	34.5
Primary	41	16.1
JSS/Middle school	43	16.9
SSS/Vocational school	39	15.3
Tertiary	43	16.9
Others	1	0.4
**Religion**		
Christianity	208	81.5
Islamic	42	16.5
Others	5	2.0
**Currently staying with family member**		
Yes	217	85.1
No	38	14.9

*Source*: Authors’ own work

* Mean (SD); Min/Max 38; 17/60

### Percentage distribution of attitudes of health service providers narrated by people with disabilities themselves

**FIGURE 1:**
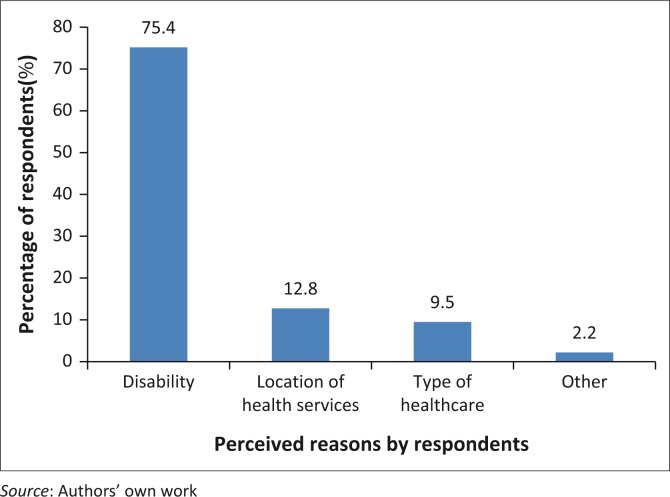
Basis for discrimination against people with disabilities.

**FIGURE 2:**
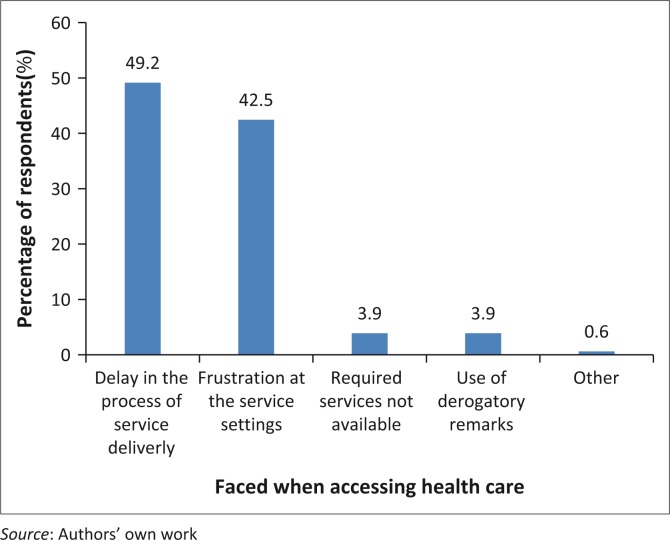
Forms of discrimination against people with disabilities.

Table 2 and Figures 1–2 present results on the perceived attitudes of health providers towards people with disabilities at the health facility. On the question whether people with disabilities faced discrimination when they accessed health care, the majority of respondents (71%) disclosed they did, whereas the remained had a contrary opinion. On their ability to ask questions about treatment, 63% of respondents admitted that the service providers allowed them to ask questions when they did not understand treatment concerning their health care. Again, the majority (58.8%) of respondents disclosed that service providers had enough time to explain their health condition for them to understand, whereas 41.2% admitted not to have experienced such a situation. More than 50% of respondents perceived the service providers’ knowledge on disability issues as limited.

**TABLE 2:** Percentage distribution of perceived attitudes of health care providers towards People with disabilities.

Variables	Frequency	Percentage
**Face discrimination when accessing health care**		
Yes	181	71.0
No	74	29.0

**Total**	**255**	**100**

**Service providers allow People with disabilities to ask when they do not understand something about the medical process**		
Yes	162	63.5
No	93	36.5

**Total**	**255**	**100**

**Service providers have enough time for People with disabilities to explain the medical process for understanding**		
Yes	150	58.8
No	105	41.2

**Total**	**255**	**100**

**Rating of health care providers knowledge on disability issues**		
Very good	15	5.9
Good	90	35.3
Very bad	18	7.1
Bad	132	51.8

**Total**	**255**	**100**

*Source*: Authors’ own work

As shown in Figure 1, respondents who faced discrimination gave reasons which were largely based on their disability (75.4%). Respondents (12.8%) were also of the view that the physical location of the health facility away from their residency discriminated against them. Figure 2 also demonstrates the forms of discrimination that people with disabilities faced when accessing health care. Most of the respondents experienced delays in the process of health care delivery (49.2%), frustration from long waiting time at health care settings (42.5%), derogatory remarks (3.9%) and required services being not available. Another 0.6% of respondents disclosed that health professionals were unconcerned when being treated at hospitals.

In Table 3, the influence of socio-demographic information on discrimination against people with disabilities is explored. There was an increase of the odds of being discriminated with the community of residents. Those staying in Subin were 4.64 times more likely to be discriminated against compared with those staying in Oforikrom whilst those staying in Tafo were 2.85 times more likely to face discrimination at the facility compare to those in Oforikrom. Women were more likely to face discrimination (OR = 2.40, 95% CI; 1.63, 3.52) compared with men. The odds that respondents faced discrimination increases with age. Those in the age group 31–40 were 2.56 times more likely to face discrimination compared with those below 30 years, whilst those between the ages of 41–50 were 3.33 times more likely to be discriminated at the facility compared to those below the age of 21 years. Respondents with visual impairment were 2.14 times more likely to face discrimination compared with physical impaired persons. Meanwhile, respondents who had some level of education were less likely to face discrimination at the facility compared with those with no formal education. People with disabilities who stayed with a family member at the time of the study were less likely to face discrimination at the facility (OR = 0.20, 95% CI; 0.09, 0.41).

**TABLE 3:** Logistic regression analysis of socio-demographic factors on perceived discrimination against people with disabilities.

Variables	Perceived discrimination at health facility					

	OR	95% CI	p-value	AOR	95% CI	p-value
**Community**						
Oforikrom	1.00	-	-	1.00	-	-
Subin	4.64	1.66, 12.98	<0.01	24.25	5.2, 112.26	<0.01
Asawase	1.26	0.55, 2.87	0.57	0.57	0.13, 2.48	0.46
Tafo	2.85	1.16, 6.96	<0.01	5.66	1.33, 24.06	<0.01
Asokwa	0.80	0.36, 1.79	0.59	4.60	1.05, 20.12	<0.05	**Gender**						
Male	1.00	-	-	1.00	-	-
Female	2.40	1.63, 3.52	<0.01	3.89	1.41, 10.76	<0.01
**Age**						
≤20	1.00	-	-	1.00	-	-
21–30	1.70	0.93, 3.10	0.08	3.37	0.52, 21.53	0.19
31–40	2.56	1.68, 3.91	<0.01	7.69	1.22, 48.33	<0.05
41–50	3.33	1.69, 7.44	<0.01	3.08	0.57, 16.41	0.18
>50	2.64	1.42, 4.88	<0.01	14.32	2.02, 101.51	<0.01
**Disability type**						
Physical	1.00	-	-	1.00	-	-
Blind	2.14	1.36, 3.39	<0.01	5.05	1.44, 17.65	<0.05
Deaf	1.00	-	-	-	-	-
**Employment**						
Unemployed	1.00	-	-	1.00	-	-
Government	2.99	1.05, 8.50	<0.01	1.02	0.10, 9.92	0.98
Trading	3.06	1.24, 7.58	<0.01	0.59	0.10, 3.37	0.56
Farming	3.90	1.26, 12.07	<0.01	1.00	-	-
Apprenticeship	1.45	0.72, 2.91	0.29	6.76	1.19, 38.25	<0.05
**Educational Level**						
No education	1.00	-	-	1.00	-	-
Basic education	0.16	0.06, 0.37	<0.01	0.01	0.002, 0.11	<0.01
Secondary	.015	0.05, 0.42	<0.01	0.005	0.00, 0.06	<0.01
Tertiary	0.07	0.02, 0.20	<0.01	0.04	0.006, 0.28	<0.01
**Religion**						
Christianity	1.00	-	-	1.00	-	-
Islamic	1.33	0.72, 2.45	0.35	0.34	0.10, 1.17	0.09
**Staying with family**						
No	1.00	-	-	1.00	-	-
Yes	0.20	0.09, 0.41	<0.01	0.08	0.01, 0.39	<0.01

*Source:* Authors’ own work

OR, Odds ratio; AOR, Adjusted odds ratio; CI, confidence interval, Outcome measures: Perceived discrimination at health facility.

Consistently, people staying in Subin and Tafo respectively were 5.2 times and 1.33 times more likely to face discrimination at the facility after the inclusion of other co-variates. Also, women were 3.89 times more likely to face discrimination; AOR 3.89 (95% CI; 1.41, 10.76), after accounting for the effect of other confounding variables. Different age groups were consistently associated with being discriminated after adjusting for other co-variates. Consistently, visually impaired people were more likely to be discriminated at the facility compared with physical disability after the inclusion of other co-variates; AOR = 5.05 (95% CI; 1.44, 17.65). Again, respondents with some educational qualification were less likely to face discrimination at the facility compared with those with no formal education after adjusting for other co-variates. Respondents who stayed with their family members were consistently less likely to face discrimination compared with those who were not staying with their family members after the inclusion of other co-variates; AOR = 0.08 (95% CI; 0.01, 0.39).

## Discussions

Awareness of disability issues has gained considerable interest by advocacy groups in recent years. However, it is uncertain whether attitudes and perceptions of all service providers and society have adjusted accordingly towards the health care of people with disabilities. This article sought to examine the attitudes of health care providers from the perspectives of people with disabilities in the Kumasi Metropolis in Ghana. This study found that most people with disabilities self-reported that they faced discrimination when they accessed health care. This suggests that health professionals might not have much experience seeing patients with disabilities. This finding corroborates previous studies where discrimination by health professionals limit people with disabilities from accessing health care compared to the non-disabled (Iezzoni 2011; Jones *et al*. 2008). The finding that people with disabilities faced discrimination further confirms previous findings where attitudes and behaviour of primary health care providers were barriers for people with disabilities as they seek health care (Jones *et al*. 2008), In this study, delays in the process of services delivery, the use of derogatory remarks, frustration from long waiting times at health care centers and unavailable required services were the types of discrimination reported by people with disabilities. In a previous study, discrimination was, however, commonly related to stigma and the use of abusive words (Iezzoni 2011).

The study found women to be more subjected to discrimination at the facility than males. This could be attributed to the fact that most societies have cultural preferences for males (United Nations 2003). Some cultures perceive it as a waste of resources to help disabled women to become productive which consequently relegate them to the last priority. This finding might suggest that women with disabilities may, indeed, tend to stay away from health care to avoid repetition of experiencing stigma and discrimination as reported by Fiduccia and Wolfe (1999). Again, the visually impaired were found to be the group more likely to face discrimination at the facility compared those with a physical disability. This finding implies that the varied needs of the visually impaired when accessing health care could incite health professionals to develop hatred and discrimination. Despite the differences in discrimination, it was evident that respondents with some level of education were less likely to face discrimination at the facility compared with those with no formal education. This might indicate the respect health professionals have for educated individuals in the Ghanaian society. The study found that respondents who stayed with their family members were consistently less likely to face discrimination, suggesting that the family members are likely to provide support at the facility and reduce the burden on the health professionals. Although discrimination could either be experienced by both people with disabilities and the non-disabled, most people with disabilities perceived that they faced discrimination to health care based on their disabilities, type of health care accessed as well as the location of the services. This finding reinforce that people with disabilities might indeed face discrimination (Iezzoni 2011).

The discrimination experienced by respondents demonstrates the level of exclusion when accessing health care. The finding has the implication that the services offered to patients with disabilities are not friendly and make them feel disconnected from the health system. Some health professionals appear not to have time and feel uncomfortable attending to clients with disabilities. The findings further confirm the notion that service providers might not have enough knowledge on disability issues. The perceived low level of service providers’ knowledge on disability issues may influence their attitudes towards people with disabilities’ health care as reported by Jones *et al*. (2008). Discriminatory attitudes, coupled with inadequate knowledge on disability issues amongst providers, may also lead to serious health disparities amongst people with disabilities as reported earlier in the study by Iezzoni (2011).

The observations established in this study reaffirm the need for improving issues and rights of people with disabilities in curricula for all service providers to avoid unnecessary discrimination against them. A study by Kroll *et al*. (2006) has also suggested regular education on disability issues for health service providers. This could resolve some attitudinal barriers to health care amongst people with disabilities.

This study found most people with disabilities attesting to the fact that service providers allow them to ask questions when they do not understand something concerning their health care. Findings further suggest that service providers have enough time, and explain medical issues to people with disabilities. Unlike the general population, people with disabilities require special attention at the facility. The realisation that professionals have time to respond to every question asked by people with disabilities will help to improve their health care. The finding also implies that although providers may have enough time for people with disabilities but, because of the discrimination attitudes exhibited, they may not value the time. It is necessary that the health professionals exhibit positive attitudes towards people with disabilities at the health facility.

## Conclusion

The study concludes that people with disabilities face discrimination at health care settings, such as delays in the process of service delivery, use of derogatory remarks against them, frustration and unavailability of service. It was evident that people with disabilities faced discrimination based on their disability, type of health care and the location of the service. The perceived discrimination experienced by people with disabilities at the health facility varied in terms of the socio-demographic information. The study concludes that women and visually impaired persons were the groups likely to be discriminated at the health facility; however, educated persons were less subjected to discrimination. This requires the need to invest much more into the education of disabled people. The study further concludes that people with disabilities in the Kumasi Metropolis perceived health care providers to have limited knowledge on disability issues. Providers have, however, enough time to explain medical conditions to people with disabilities and allow them to ask questions concerning their health care. The study has an implication for policy actions to change the attitudes and knowledge level of professionals. It can therefore be concluded that in-service training should be organised for service providers to always update their knowledge on disability-related issues.
